# Single-Cell Transcriptome of Wet AMD Patient-Derived Endothelial Cells in Angiogenic Sprouting

**DOI:** 10.3390/ijms232012549

**Published:** 2022-10-19

**Authors:** Natalie Jia Ying Yeo, Vanessa Wazny, Nhi Le Uyen Nguyen, Chun-Yi Ng, Kan Xing Wu, Qiao Fan, Chui Ming Gemmy Cheung, Christine Cheung

**Affiliations:** 1 Lee Kong Chian School of Medicine, Nanyang Technological University, Singapore 636921, Singapore; 2 Duke-NUS Medical School, National University of Singapore, Singapore 169857, Singapore; 3 Ophthalmology & Visual Sciences Academic Clinical Program (Eye ACP), Duke-NUS Medical School, Singapore 169857, Singapore; 4 Singapore Eye Research Institute, Singapore 169856, Singapore; 5 Institute of Molecular and Cell Biology, Agency for Science, Technology and Research, Singapore 138673, Singapore

**Keywords:** age-related macular degeneration, blood outgrowth endothelial cells, sprouting angiogenesis, single-cell transcriptome, endothelial cell states

## Abstract

Age-related macular degeneration (AMD) is a global leading cause of visual impairment in older populations. ‘Wet’ AMD, the most common subtype of this disease, occurs when pathological angiogenesis infiltrates the subretinal space (choroidal neovascularization), causing hemorrhage and retinal damage. Gold standard anti-vascular endothelial growth factor (VEGF) treatment is an effective therapy, but the long-term prevention of visual decline has not been as successful. This warrants the need to elucidate potential VEGF-independent pathways. We generated blood out-growth endothelial cells (BOECs) from wet AMD and normal control subjects, then induced angiogenic sprouting of BOECs using a fibrin gel bead assay. To deconvolute endothelial heterogeneity, we performed single-cell transcriptomic analysis on the sprouting BOECs, revealing a spectrum of cell states. Our wet AMD BOECs share common pathways with choroidal neovascularization such as extracellular matrix remodeling that promoted proangiogenic phenotype, and our ‘activated’ BOEC subpopulation demonstrated proinflammatory hallmarks, resembling the tip-like cells in vivo. We uncovered new molecular insights that pathological angiogenesis in wet AMD BOECs could also be driven by interleukin signaling and amino acid metabolism. A web-based visualization of the sprouting BOEC single-cell transcriptome has been created to facilitate further discovery research.

## 1. Introduction

Age-related macular degeneration (AMD) is a major cause of irreversible blindness in older populations [1,2]. An exudative subtype of this disease, known as ‘wet’ AMD, is responsible for approximately 90% of AMD cases with severe vision loss [3]. Wet AMD is characterized by the anomalous vascularization of choroidal vessels into the subretinal space (choroidal neovascularization) with accompanying exudate accumulation and/or hemorrhage leading to vision loss [4,5,6]. During disease development, degenerative changes occur in the retinal pigmented epithelium, choroidal vessels, Bruch’s membrane, and photoreceptor layers that ultimately create a proinflammatory and angiogenic milieu favoring choroidal neovascularization [7]. Anti-vascular endothelial growth factor (VEGF) treatment is the gold standard therapy for wet AMD [8,9]. However, anti-VEGF treatment is effective in a subset of AMD patients and seems ineffective in the long-term prevention of visual decline [8,10,11,12]. Such findings warrant a need to illuminate its deeper molecular mechanisms, as well as VEGF-independent pathways.

In vivo models have been crucial to shed light on the molecular pathogenesis of wet AMD. A common animal model for wet AMD is the laser-induced choroidal neovascularization model, in which neovascularization of choroidal vessels in the retina is induced by laser exposure to the Bruch’s membrane [10,13]. While these murine models have provided valuable insights into mechanisms and therapeutic testing [13,14,15], the mouse eye is lacking a defined macula compared to humans [10,16,17]. Laser-induced choroidal neovascularization models may not fully capture the genetic and pathological complexities of human wet AMD [13,18]. On the other hand, numerous in vitro human-relevant models of wet AMD have been created from retinal pigmented epithelium [19,20,21]. Despite the involvement of choroidal vascular endothelium in the pathogenesis of both typical wet AMD and other clinical variants [7,22,23,24,25], few patient-derived vascular cell models of wet AMD have been developed for disease interrogation [26,27,28,29,30]. There remain knowledge gaps in the investigation of endothelial-specific mechanisms in wet AMD.

To elucidate the disease molecular signatures of human wet AMD endothelium during angiogenesis, we leveraged on our model of blood outgrowth endothelial cells (BOECs) developed from wet AMD patients. We and others have shown that BOECs can be derived from peripheral blood of patients and established as endothelial models recapitulating the morphological and functional characteristics of mature endothelial cells [31,32,33,34,35]. Here, we studied angiogenic behaviors of patient BOECs using a three-dimensional sprouting assay. As sprouting angiogenesis induces multiple endothelial phenotypes [36], we performed analysis on the single-cell RNA-sequencing (scRNA-seq) of sprouting BOECs to resolve the endothelial subpopulations. To facilitate data mining of wet AMD endothelial molecular hallmarks, we created a web-based visualization of sprouting BOEC single-cell transcriptome (https://christinecheunglab.shinyapps.io/human_wet_AMD_sprouting/, accessed and last updated on 10 October 2022).

## 2. Results

**Figure 1:**
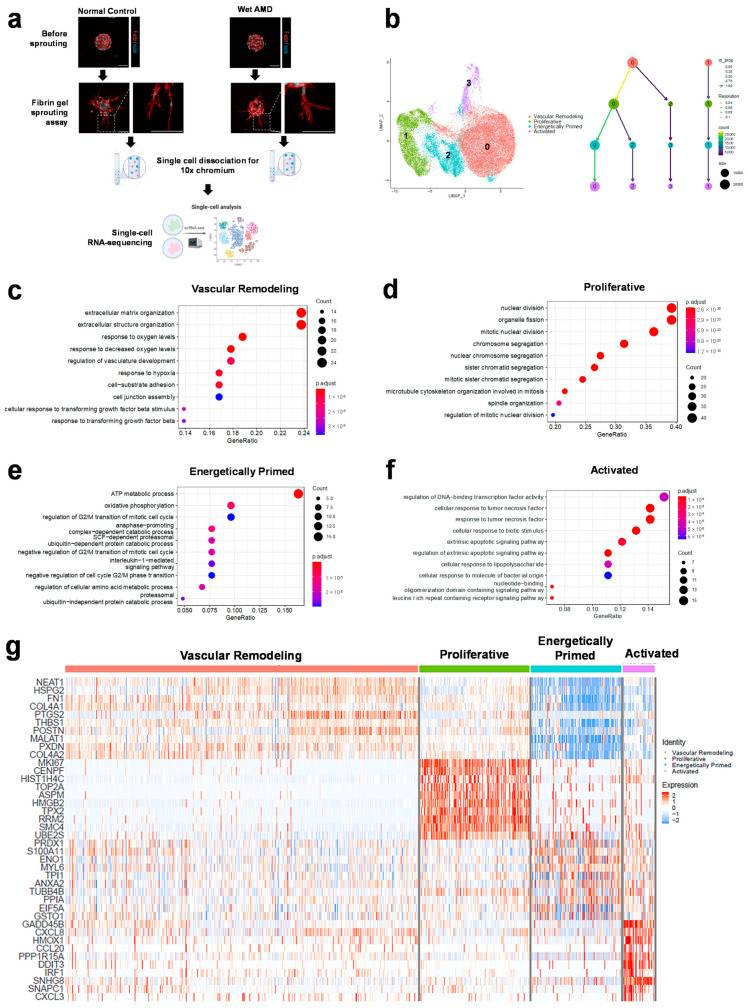
Human blood outgrowth endothelial cells adopt distinct cell states during sprouting angiogenesis. (**a**) Blood outgrowth endothelial cells (BOECs) derived from wet AMD and normal subjects were induced to sprout from cell-coated microcarrier beads in a fibrin gel sprouting angiogenesis assay, dissociated into single cell suspensions and subjected to single-cell RNA sequencing for downstream analysis. Images show confocal micrographs of representative normal and AMD BOECs at 24 h of the sprouting assay (red: f-actin; cyan: nuclei). Scale bar, 100 µm. (**b**) UMAP of integrated dataset showing derived endothelial clusters (**left**) and clustering tree depicting the relationships between clusters as resolution increases from 0.04 to 0.1 (**right**). Size of nodes (size) represents number of cells per cluster and color of nodes (Resolution) the resolution used to generate clusters. Numbers in each node represent cluster names in their corresponding resolutions. Edges are colored according to the number of cells from the incoming node (count). (**c**–**f**) Enrichment analysis of the top 100 cluster markers sorted by log2 fold change based on the Gene Ontology Biological Processes knowledgebase. Only top 10 enriched processes based on adjusted *p* values (p.adjust) are shown. Count indicates the number of genes within each term. (**g**) A heatmap of top 10 marker genes for each cluster and their corresponding expressions per cell.

We have developed our BOEC models from peripheral blood mononuclear cell fractions isolated from wet AMD patients and normal controls [31]. Demographics of the donors from whom BOECs were used here have been included in Supplemental Table S1. These BOECs were subjected to a three-dimensional fibrin gel bead sprouting assay for 24 hours, displaying multiple sprouts on the beads with filopodia (Figure 1a), which were characteristic of endothelial cells responding to angiogenic cues [37]. To deconvolute the heterogeneity of angiogenic endothelial cells from patients, we dissociated sprouting BOECs in fibrin gel using nattokinase and prepared individual scRNA-seq libraries for sequencing (Figure 1a).

### 2.1. Human Blood Outgrowth Endothelial Cells Undertake Distinct Cell States during Sprouting Angiogenesis

To determine an optimal number of endothelial cell states, we took reference of the cellular phenotypes that could arise during angiogenesis. In vascular angiogenic sprouts, at least three types of cell specializations are present—tip cells that are the leading cells of the vascular sprout typically showing filopodia extensions, stalk cells that trail after tip cells and display active proliferation, and endothelial cells of the main vascular plexus [37]. We generated a clustering tree for 4 resolutions ranging from 0.04 to 0.1 using Seurat’s *FindClusters* function (Figure 1b, right panel). Based on the clustering hierarchy, Clusters ‘0′ and ‘1′ were transcriptomically distinct and represented the primary populations. At higher resolutions (0.08 and 0.1), Cluster ‘1′ remained independent from the other clusters, while Cluster ‘0′ diverged into Cluster ‘3′ and subsequently gave rise to Cluster ‘2′. Both Clusters ‘2′ and ‘3′ remained stable as the evolved populations. We were cognizant that tip and stalk cell specializations are transient and interchangeable, depending intricately on molecular cues (local VEGF, Notch signaling) [38,39,40]. Four transcriptomic clusters (Figure 1b, left panel) might reflect the dynamism of angiogenic phenotypes and potentially recapitulate a continuum of transitory cell states in the endothelial sprouts.

Upon derivation of cluster marker genes and functional enrichment analysis of these cluster markers (Figure 1c–f), we assigned the four endothelial cell states, Clusters ‘0′, ‘1′, ‘2′ and ‘3′, as vascular remodeling, proliferative, energetically primed and activated, respectively. Vascular remodeling is a process by which blood vessels restructure and rearrange through cell migration, cell growth, and degradation or synthesis of the extracellular matrix [41]. Under the gene ontology term ‘response to hypoxia’ (Figure 1c), vascular remodeling BOECs expressed genes such as VEGF-induced *PTGS2* (cyclooxygenase-2), an early response gene that leads to downstream proangiogenic effects [42,43], *THBS1* (thrombospondin-1), a hypoxia-induced modulator of vascular remodeling in pulmonary arterial hypertension [44,45], and *EDN1* (endothelin-1), a potent growth factor that induces the migration, proliferation and invasion of endothelial cells during angiogenesis [46]. The other enriched processes of ‘cell-substrate adhesion’ and ‘extracellular matrix organization’ (Figure 1c) emphasized matrix reorganization during cell migration. Substantiating the vascular remodeling phenotype of BOECs in this cluster, molecular functions such as ‘extracellular matrix structural constituent’ (consisting of collagen genes) and ‘integrin binding’ (Supplementary Figure S1a) suggested that vascular remodeling BOECs might be participating in the initiation of angiogenesis.

Another distinct primary population, the proliferative BOECs displayed many enriched biological processes relating to mitotic cell division and microtubule binding (Figure 1d and Supplemental Figure S1b). Among the top marker genes of proliferative BOECs (Figure 1g), *MKI67*, *CENPF*, and *TOP2A* are well-known proliferation markers and *ASPM* encodes a mitotic spindle protein [47,48]. It is plausible that these proliferative BOECs present in the fibrin gel sprouting assay were inherently in the dividing phase of the cell cycle or as part of vascular sprout formation [37].

Energetically primed BOECs had evolved from the vascular remodeling BOECs. The enriched processes ‘ATP metabolic process’ and ‘oxidative phosphorylation’ (Figure 1e) revealed that these energetically primed BOECs were switching out from a quiescent state to undergo the most energetically demanding phase of angiogenesis. The increased expression of cluster markers *ENO1* (enolase 1) and *TPI1* (triosephosphate isomerase 1) (Figure 1g), both glycolytic enzymes [49,50], suggested that glycolytic pathways were also prevalent in these cells. Glycolytic metabolism is increased in angiogenic endothelial cells to generate energy for either cell motility or proliferation [51]. In line with this, it has been reported that VEGF can stimulate both glycolysis and mitochondrial respiration in ECs in vitro [52]. Interestingly, energetically primed BOECs also demonstrated the biological process ‘negative regulation of G2/M transition of the cell cycle’ (Figure 1e), which was mainly represented by genes encoding for proteasomal subunits. The degradation of cyclins and other cell cycle regulators by the 26S proteosome is essential to the oscillation of cell cycle proteins and consequently regulation of the cell cycle [53,54]. Specifically, as indicated by the biological processes ‘SCF-dependent proteasomal ubiquitin-dependent protein catabolic process’ and ‘anaphase-promoting complex-dependent catabolic process’ (Figure 1e), these BOECs seemed to involve the ubiquitin ligases SCF complex, which degrades G1 Cdk inhibitors to control entry into S phase, and anaphase-promoting complex, which is essential for exit from mitosis [54]. Inactivation of the anaphase-promoting complex also enables its substrates the glycolytic enzyme PFKFB3 and glutaminase to upregulate glycolysis and the biosynthesis of macromolecules for cell cycle progression [55,56], therefore linking the upregulation of metabolic processes with cell cycle regulation observed in energetically primed BOECs.

Activated BOECs might have temporally developed from the vascular remodeling BOECs. Biological processes such as ‘cellular response to tumor necrosis factor’ and ‘cellular response to lipopolysaccharide’ (Figure 1f), as well as increased gene expressions of NFκB pathway mediators, were characteristic of activated endothelium [57]. Tumor necrosis factor signaling could be inducing the specialization of tip cell-like phenotypes in this population [58]. Furthermore, molecular function analysis revealed ‘cytokine receptor binding’ and ‘chemokine activity’, suggesting that activated BOECs were actively involved in binding of inflammatory molecules and stress response (Supplemental Figure S1d). After determining the overarching cell states, we proceeded to analyze wet AMD versus normal control endothelial cells.

### 2.2. Wet AMD Endothelial Cells Reveal Pathological Angiogenesis Hallmarks

**Figure 2:**
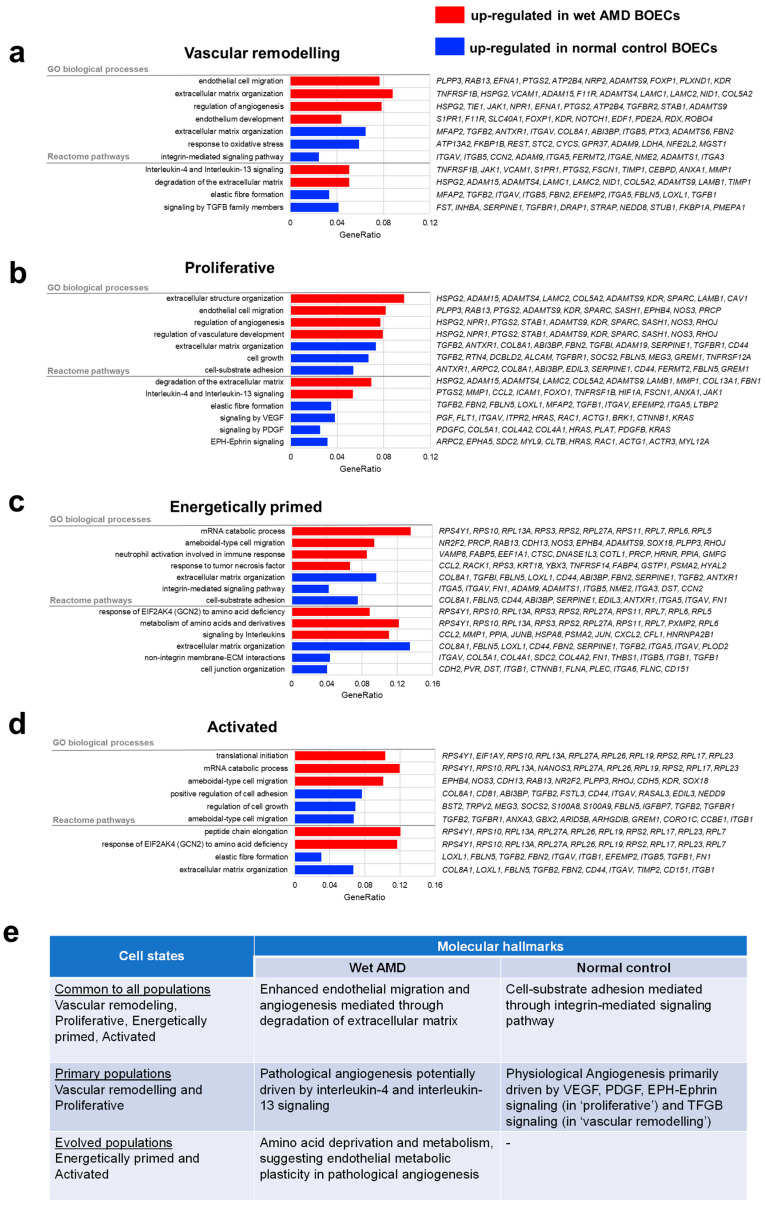
Wet AMD endothelial cells reveal pathological angiogenesis hallmarks. (**a**–**d**) Enrichment of top 100 differentially expressed genes (p. adj < 0.05) based on gene ontology biological processes and Reactome Pathway knowledgebases, for each of the four clusters as indicated. The top enriched processes and pathways are shown. Red bars represent wet AMD BOEC-upregulated processes, while blue bars represent normal BOEC-upregulated processes. (**e**) Summary of molecular hallmarks comparing wet AMD and normal BOECs that are common to all populations and found in primary/evolved populations.

Next, we performed differential expression analysis within each population to compare the transcriptomic profiles of wet AMD and normal sprouting BOECs. We found that across all populations, wet AMD BOECs consistently featured endothelial cell migration and regulation of angiogenesis, while normal BOECs demonstrated cell-substrate adhesion (Figure 2a–d). In the primary populations, extracellular matrix organization and/or degradation were upregulated pathways in wet AMD BOECs (Figure 2a,b), suggesting that matrix degradation mediated their enhanced migration and angiogenesis. Since early stages of angiogenesis require the degradation of extracellular matrix for migration and proliferation of vascular sprouts [59], wet AMD BOECs could adopt a more angiogenic and migrative phenotype than normal control cells. Normal BOECs, on the other hand, reflected cell-substrate adhesion as a consistent feature across the proliferative, energetically primed and activated populations (Figure 2b–d). The process of cell substrate adhesion seemed to be mediated by ‘integrin signaling pathways’ that included genes such as *ITGA3*, *ITGA4*, *ITGAV* and *ITGB5* (Figure 2c). Specific integrins are known to be upregulated in endothelial cells during VEGF/TGFβ-mediated angiogenesis [60,61]. Both α_v_β_3_ and α_v_β_5_ are among the key mediators of physiological angiogenesis. Ablation of β_3_ and β_5_ integrins actually resulted in increased pathological angiogenesis and tumor growth [62], indicating that normal BOECs possessed integrins that were unlikely the drivers of neovascularization under pathological states.

Focusing on the primary populations—vascular remodeling and proliferative BOECs, wet AMD BOECs had upregulated ‘interleukin-4 and interleukin-13 signaling’ (Figure 2a,b). In line with these findings, concentrations of interleukin-4 (IL-4) and interleukin-13 (IL-13) in serum and aqueous humor samples were significantly elevated in AMD patients compared to healthy controls [63,64,65]. The receptor of IL-4, IL-4Rα, stimulated pathological angiogenesis of bone marrow-derived endothelial progenitor cells, contributing to choroidal neovascularization formation [65]. Furthermore, both IL-4 and IL-13 induced mRNA expression of *VCAM1* in vascular endothelial cells which is involved in VCAM-1/integrin α_4_ adhesion during inflammation and angiogenesis [66]. Supporting this notion, *VCAM1* was one of the wet AMD-upregulated genes in the primary populations (Figure 2a,b). These data suggest that IL-4 and IL-13 signaling may be responsible for enhanced pathological angiogenesis, independent of VEGF, in wet AMD BOECs.

Normal BOECs in the proliferative population appeared to be driven by ‘signaling by VEGF’, ‘signaling by PDGF’ and ‘Eph-Ephrin signaling’ (Figure 2b). Firstly, among the upregulated pathway ‘signaling by VEGF’ in normal BOECs, *FLT1* is a decoy receptor binding with high affinity to VEGFA but acts to suppress VEGF signaling in the vascular endothelium [67]. During tip-stalk competition in angiogenic sprouting, cells that express lower FLT1 levels gain the advantage of becoming and maintaining the tip cell phenotype [39]. Correspondingly, the downregulated *FLT1* in wet AMD BOECs might favor excessive tip cell formation. Secondly, upregulated Eph/Ephrin signaling in normal BOECs is responsible for modulating angiogenesis and arterio-venous remodeling to form proper capillary networks [68,69]. Some ephrins, such as *ephrinA1*, are also negative regulators of proliferation [70]. Therefore, reduced ephrin signaling in wet AMD BOECs might exert a stimulatory effect on proliferation, contributing to their proangiogenic phenotype.

Moving onto the evolved populations—energetically primed and activated BOECs, wet AMD BOECs had upregulated pathways such as ‘response of *EIF2AK4* (*GCN2*) to amino acid deficiency’ and ‘metabolism of amino acids and derivatives’ (Figure 2c,d). *EIF2AK4* is a sensor of amino acid starvation that induces ATF4 signaling resulting in the secretion of angiogenic VEGF by vascular endothelial cells [71]. We postulated that wet AMD BOECs experienced amino acid starvation, thus activating *EIF2AK4* to increase VEGF production. Interestingly, amino acid deprivation-induced VEGF expression is evident in human tumors and tumor cell lines, suggesting that the EIF2AK4/ATF4 axis stimulates tumor angiogenesis. Wet AMD BOECs in energetically primed population also upregulated genes for amino acid metabolism (Figure 2c). We expected that energetically primed BOECs could gain additional biomass and energy during angiogenic sprouting. To increase biomass, proliferating/migrating endothelial cells increased glycolytic flux, fatty acid metabolism and amino acid metabolism compared to quiescent endothelial cells [72]. Furthermore, it has been reported that the switch from quiescence to angiogenic endothelial states in mouse choroidal neovascularization was accompanied by metabolic transcriptome plasticity [73]. Hence, wet AMD BOECs might have upregulated amino acid metabolism pathways to meet biomass demands in their enhanced propensity to undergo hypersprouting. We have distilled the molecular hallmarks comparing wet AMD and normal BOECs in Figure 2e.

### 2.3. Benchmarking Human Sprouting BOECs to In Vivo Choroidal Neovascularization Profiles

**Figure 3:**
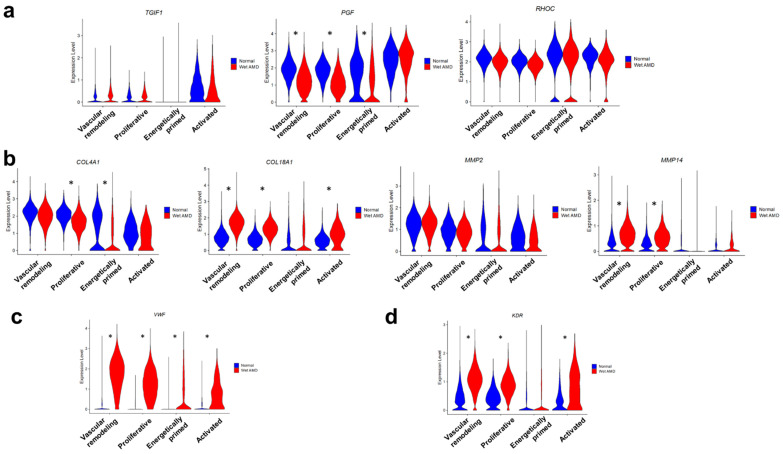
Benchmarking of human sprouting BOECs to in vivo choroidal neovascularization profiles. (**a**) Violin plots showing expressions of curated CNV tip endothelial cell markers based on Rohlenova et al. (**b**) Violin plots showing expressions of collagen genes and matrix metalloproteinases. (**c**) Violin plot showing expression of *VWF*, an injury marker, in normal and wet AMD sprouting BOECs. (**d**) Violin plot showing expression of *KDR*, a VEGF receptor, in normal and wet AMD sprouting BOECs. Asterisks mark significant differential expression between wet AMD and normal BOECs within each cluster, as determined using *FindMarkers*.

In benchmarking our human endothelial angiogenic model to the gold standard laser-induced choroidal neovascularization (CNV) model, we studied Rohlenova et al.’s transcriptome profiling of endothelial cells in murine CNV [73]. We recognized that our wet AMD BOECs subpopulations might not converge exactly to the distinct phenotypes of murine CNV in vivo. However, there were some resemblances of our human BOEC cell states to the in vivo tip endothelial cells, proliferating endothelial cells and angiogenic endothelial cells of post-capillary venule origin, etc. We curated a few markers from the CNV tip endothelial cells, namely *TGIF1*, *PGF* and *RHOC*. When we visualized these genes in our BOEC dataset, activated BOECs appeared to express relatively higher levels of *TGIF1* and *PGF* than the other populations, even though we did not resolve major differences between wet AMD and normal BOECs (Figure 3a). *TGIF1* (TG-interacting factor 1) is a regulator of stress-induced proinflammatory phenotype in human vascular endothelial cells [74], which was in consensus with the proinflammatory tumor necrosis factor signaling in activated BOECs, potentially inducing the specialization of tip cell-like phenotypes in this population.

Collagen biosynthesis was an upregulated pathway in angiogenic endothelial cells of CNV [73]. We analyzed collagen genes, *COL4A1* and *COL18A1*, and matrix metalloproteinases, *MMP2* and *MMP14* (Figure 3b). The expressions of these targets were generally higher in the primary populations compared to the evolved populations, supporting their involvement in vascular remodeling and cell proliferation. In particular, the expression levels of *COL18A1* and *MMP14* were elevated in wet AMD BOECs compared to their normal counterparts (Figure 3b). COL18A1 (C-terminal fragment being endostatin) is a heparan sulphate proteoglycan localized to endothelial basement membrane [75,76]. Overexpression of *COL18A1* in mice leads to ultrastructural changes, disorganization and loosening of the basement membrane [77], while upregulation of *MMP14* contributes to a change in extracellular matrix composition/stiffness that promotes a more angiogenic phenotype of endothelial cells [78,79]. Taken together, *COL18A* or *MMP14* might confer abnormal changes to the extracellular matrix integrity in wet AMD BOECs.

Notably, another CNV-associated gene, *VWF*, was pronouncedly increased in wet AMD BOECs than normal BOECs (Figure 3c). VWF is a well-known endothelial activation marker and is produced in response to mediators such as cytokines, epinephrine or vasopressin [80]. Plasma levels of vWF is also increased in wet AMD patients compared to controls [81,82]. We hypothesized that our wet AMD BOECs might recapitulate some of the hallmarks of injury response in patients. Wet AMD BOECs also showed the same trend by having higher expression of *KDR*, the vascular endothelial growth factor receptor 2, than normal BOECs (Figure 3c). Trafficking of intracellular KDRs to the cell surface increases the sensitivity of endothelial cells to VEGFA [83]. Therefore, wet AMD BOECs might have increased sensitivity to angiogenic stimuli.

## 3. Discussion

We analyzed a single-cell transcriptomic dataset of wet AMD and normal control endothelial cells that had been induced to undergo angiogenic spouting. Our human sprouting BOEC model shares some common traits with the murine laser induced CNV model [73]. Firstly, we found that ‘activated’ BOECs showed proinflammatory hallmarks and were transcriptomically more like the tip-like cell population in CNV in vivo. CNV in wet AMD is known to be associated with an inflammatory milieu, involving cytokines and complement system activation [84,85]. Secondly, extracellular matrix regulation of endothelial phenotypes was apparent in our wet AMD BOECs and CNV in vivo. Perturbations to collagen biosynthesis and turnover could induce basement membrane changes that promote proangiogenic activity of endothelial cells. Finally, wet AMD BOECs possessed markers of increased sensitivity to angiogenic and other stress paradigms. Transcriptomic analyses of human tissue with cerebrovascular malformations, such as cerebral cavernous malformations and brain arteriovenous malformations, have similarly identified enriched pathways associated with angiogenesis, including endothelial cell migration and extracellular matrix remodeling [86,87,88].

We have also provided new insights that were previously not known to be implicated in CNV. Angiogenesis in our wet AMD BOECs was driven by interleukin signaling and amino acid metabolism. While these processes have not been well reported in the pathogenesis of CNV, our wet AMD BOECs reflected pathological angiogenesis mechanisms observed in other diseases with vasculopathy or aberrant vessel growth, such as in tumor angiogenesis [89,90,91]. On the other hand, the normal control BOECs featured physiological angiogenesis mediated by VEGF, PDGF and Eph-Ephrin signaling. The main disparity of our BOEC model from the mouse CNV model is that laser induced CNV stimulates acute angiogenic response [13,18], whereas our patient-derived BOECs can capture the human genetic complexity and retain some age-related disease hallmarks in vitro [31,32,92,93].

We acknowledge the limitations of this study, including a small sample size of donors and lack of functional validation. The derivation of human BOECs does not involve organotypic differentiation for the cells to behave exactly like choroidal endothelial cells. Nevertheless, our study has elucidated a few interesting endothelial processes that may underpin pathological angiogenesis in wet AMD, warranting further investigations. To this end, we have created an interactive web-based visualization of the sprouting BOEC dataset (https://christinecheunglab.shinyapps.io/human_wet_AMD_sprouting/, accessed and last updated on 10 October 2022), to provide a transcriptomic resource for discovery research.

## 4. Materials and Methods

### 4.1. Patient Selection and Sample Collection

Participants (2 wet AMD and 1 normal subjects) were enrolled from the retina clinic of the Singapore National Eye Center. Inclusion criteria were age 40–80 years (demographics detailed in Supplemental Table S1). Participants with wet AMD were specifically diagnosed to have polypoidal choroidal vasculopathy (PCV) based on the presence of polypoidal dilatations in indocyanine green angiography (ICGA). A healthy volunteer was selected for the control group based on the lack of AMD or PCV from clinical examination. To collect blood samples for downstream analysis, 10 mL of fresh blood specimen was obtained from each participant and processed in the laboratory within 6 h. Upon Ficoll^®^ Paque (GE Healthcare, Chicago, IL, USA) centrifugation of the blood sample, the resulting buffy coat layer which contained peripheral blood mononuclear cells (PBMCs) was isolated from which DNA extraction was performed for genotyping with the OmniExpress chip. The remaining PBMCs were cultured for the derivation of blood outgrowth endothelial cells (BOECs).

### 4.2. Derivation and Maintenance of Blood Outgrowth Endothelial Cells

BOECs were generated as previously described [94] with modifications. One volume of peripheral blood samples (5–9 mL per participant) was diluted with 1 volume of phosphate-buffered saline (PBS) and the buffy coat layer was separated via density gradient centrifugation with Ficoll^®^ Paque (GE Healthcare). The buffy coat, which was enriched with peripheral blood mononuclear cells (PBMCs), was carefully collected, washed with PBS, resuspended in heparin-free, EGM-2 medium (Lonza, Basel, Switzerland) supplemented with 16% defined fetal bovine serum (FBS; Hyclone, Logan, UT, USA) and counted. Plasma was also collected and stored at −80 °C. Subsequently, the PBMCs were seeded into collagen I-coated well(s) according to a cell density of ≥1.5 × 10^6^ cells/cm^2^. Medium was replaced every two to three days. Outgrowth endothelial colonies should emerge visually between seven to 14 days after seeding. The cells were expanded to the third passage before any applications were performed, including phenotyping and functional evaluation, to opt out unwanted leukocytes. After the third passage, BOECs were cultured on collagen-I-coated tissue culture dishes in heparin-free, EGM-2 with 10% heat-inactivated FBS and medium was replaced every two to three days.

### 4.3. Fibrin Gel Bead Sprouting Assay

To subject BOECs to a sprouting context, a fibrin gel bead sprouting assay was performed as per described [95] with modifications. BOECs were coated onto Cytodex 3 microcarrier beads (Merck, Germany) at 150 cells per bead with agitation for 4 h and allowed to adhere to the beads overnight. Coated beads were subsequently suspended in 2 mg/mL fibrinogen solution (Merck, Germany) at a concentration of 500 beads/mL and clotted with 0.625 U/mL thrombin (Merck, Germany). Heparin-free EGM-2 with 10% FBS (Gibco) was added to the clotted gels containing the cell-coated beads, and cells were incubated overnight at 37 °C and 5% CO_2_ for 24 h to allow sprout formation.

To process fibrin gel sprouted BOECs for single-cell RNA sequencing, gels were rinsed with 1×PBS once, dislodged with a small spatula, and agitated for 15 min at 37 °C/5% CO_2_ in 50 FU/mL of nattokinase in 1×PBS. The resulting cell/bead suspension was collected and filtered through a 37 μm mesh strainer before being washed with heparin-free EGM-2 with 10% FBS. Subsequently, beads were resuspended with trypsin and agitated for 5 min at 37 °C/5% CO_2_. The cell suspension was then filtered through a 37 μm mesh strainer again, pelleted, and resuspended in heparin-free EGM-2 with 10% FBS, before being counted for sequencing purposes.

To capture images of representative 24 h sprouting BOECs, fixing, permeabilization, staining and image acquisition of the gel-embedded BOECs were performed as described in [31]. Briefly, the gels containing sprouted BOECs were fixed with 4% paraformaldehyde, permeabilized in 0.5% Triton X-100, stained for actin filaments using TRITC-Phalloidin in 1×PBS containing 1% BSA, and stained for nuclei with 500 ng/mL DAPI. Gel-embedded BOECs were imaged using an inverted laser scanning confocal microscope (LSM800, Carl Zeiss) using a Plan-Apochromat 20x/0.80 objective lens. Two-channel Z-stack images (AF568 and DAPI) of whole beads were captured using the ZEN software (blue edition, Carl Zeiss), acquiring images of 1024 × 1024-pixel resolution from 0.6× optical zoom at Z-intervals of 1.11 µm. For each donor BOEC line, two to four of the most representative well-formed individual filopodia were captured.

### 4.4. Single-Cell RNA Sequencing and Analysis

Each donor’s BOECs were sprouted in five wells of fibrin gels, with approximately 200 beads per well. After 24 h of sprouting assay, the BOECs embedded in fibrin gels were dissociated, pooled and split into two cell suspensions for loading onto 10× Genomics Chromium Controller chip by facility personnel at Single-cell Omics Centre (SCOC), Genome Institute Singapore (GIS). Hence, each donor’s sprouting BOECs were prepared as two separate scRNA-seq libraries using Chromium Single Cell 3′ v3 Reagent Kit (10× Genomics) by SCOC GIS and the final ready-to-sequence libraries were handed over with quantification and quality assessment reports from Bioanalyzer Agilent 2100 using the High Sensitivity DNA chip (Agilent Genomics). Individual libraries were pooled equimolarly and sent for sequencing by NovogeneAIT Genomics (Singapore). Raw sequencing data was also processed by NovogeneAIT Genomics (Singapore) using CellRanger (10× Genomics) with reads mapped to the human genome assembly (GRCh38).

Using the output filtered matrix files, analysis of the single-cell RNA sequencing of the three BOEC samples was performed using the Seurat package (v 4.1.1) [96,97]. Low-quality cells were filtered by excluding cells with low number of detected genes (nGene; less than 200) and transcript counts (nUMI; less than 500) based on inspection of the spread of these parameters per sample. To exclude potential doublets/multiplets, cells with abnormally high gene number (more than 9500) were filtered out, and *DoubletFinder* (v 2.0.3) [98] (expected doublet rate of 5%, 1:10 PCs and pK of 0.06) was applied to predict and remove doublets. The *paramSweep_v3* function and mean-variance-normalized bimodality coefficient (BCmvn) were used to determine the optimal pK value for the dataset. Furthermore, we excluded cells with high percentage of mitochondrial genes (greater than 20%), a threshold based on a previously reported mitochondrial gene percentage in endothelial cells [99]. We also excluded cells with low complexity of gene expression (less than 0.775), measured by the ratio of number of genes to number of transcripts (log[nGene]/log[nUMI]), as well as cells with low percentage of ribosomal reads (less than 5%). Finally, gene-level filtering was performed to exclude genes expressed in less than three cells.

The filtered dataset was scaled and normalized using *SCTransform* on each sample, with the parameter of variables to regress set as mitochondrial gene percentage, before integration based on 3000 integration features. After running a principal component analysis (PCA) and *RunUMAP* at 1:30 dimensions, *FindNeighbours* at 1:30 dimensions and *FindClusters* at a resolution of 0.1 were used to cluster the cells in the integrated dataset. A clustering tree was also generated using the *clustree* (v 0.4.4) function [100], which depicts the relationships between clusters as the resolution increases.

### 4.5. Cluster Marker Identification and Differential Expression Analysis

To identify cluster marker genes, *FindConservedMarkers* was applied. Gene enrichment of the top 100 cluster markers sorted by log2 fold change was performed using the clusterProfiler package (v 4.2.2) [101] and ReactomePA package (v 1.38.0) [102] with the parameters pAdjustMethod = “BH”, pvalueCutoff = 0.05, and qvalueCutoff = 0.2. To perform differential expression analysis between wet AMD and normal BOECs for each cluster, *FindMarkers* using Wilcoxon Rank Sum test and log2FC threshold of 0.25 was used. The lists were further filtered to include *p.adj* values of less than 0.05. Gene enrichment of differentially expressed genes between wet AMD and normal per cluster was conducted using the clusterProfiler and ReactomePA packages.

## Data Availability

The authors declare that all data supporting the findings of this study are available within the paper and Supplemental Materials. Specifically, single-cell sequencing (scRNA-seq) dataset that support the findings of this study are available in the Gene Expression Omnibus repository, Accession number GSE213933, upon publication of this paper.

## Supplementary Materials

The following supporting information can be downloaded at: https://www.mdpi.com/article/10.3390/ijms232012549/s1.

## References

[1] Pascolini D., Mariotti S.P. (2012). Global estimates of visual impairment: 2010. Br. J. Ophthalmol..

[2] Wong W.L., Su X., Li X., Cheung C.M.G., Klein R., Cheng C.-Y., Wong T.Y. (2014). Global prevalence of age-related macular degeneration and disease burden projection for 2020 and 2040: A systematic review and meta-analysis. Lancet Glob. Health.

[3] Chappelow A.V., Schachat A.P., Nguyen Q.D., Rodrigues E.B., Farah M.E., Mieler W.F. (2010). CHAPTER 18-Neovascular age-related macular degeneration. Retinal Pharmacotherapy.

[4] Saini J.S., Corneo B., Miller J.D., Kiehl T.R., Wang Q., Boles N.C., Blenkinsop T.A., Stern J.H., Temple S. (2017). Nicotinamide ameliorates disease phenotypes in a human iPSC model of age-related macular degeneration. Cell Stem Cell.

[5] Chirco K., Sohn E., Stone E., Tucker B., Mullins R. (2017). Structural and molecular changes in the aging choroid: Implications for age-related macular degeneration. Eye.

[6] Ambati J., Fowler B.J. (2012). Mechanisms of age-related macular degeneration. Neuron.

[7] Bhutto I., Lutty G. (2012). Understanding age-related macular degeneration (AMD): Relationships between the photoreceptor/retinal pigment epithelium/Bruch’s membrane/choriocapillaris complex. Mol. Asp. Med..

[8] Fernández-Robredo P., Sancho A., Johnen S., Recalde S., Gama N., Thumann G., Groll J., García-Layana A. (2014). Current Treatment Limitations in Age-Related Macular Degeneration and Future Approaches Based on Cell Therapy and Tissue Engineering. J. Ophthalmol..

[9] Newman D.K. (2016). Photodynamic therapy: Current role in the treatment of chorioretinal conditions. Eye.

[10] Malek G., Busik J., Grant M.B., Choudhary M. (2018). Models of retinal diseases and their applicability in drug discovery. Expert Opin. Drug Discov..

[11] Rofagha S., Bhisitkul R.B., Boyer D.S., Sadda S.R., Zhang K., Group S.-U.S. (2013). Seven-year outcomes in ranibizumab-treated patients in ANCHOR, MARINA, and HORIZON: A multicenter cohort study (SEVEN-UP). Ophthalmology.

[12] Krebs I., Glittenberg C., Ansari-Shahrezaei S., Hagen S., Steiner I., Binder S. (2013). Non-responders to treatment with antagonists of vascular endothelial growth factor in age-related macular degeneration. Br. J. Ophthalmol..

[13] Pennesi M.E., Neuringer M., Courtney R.J. (2012). Animal models of age related macular degeneration. Mol. Asp. Med..

[14] Saishin Y., Saishin Y., Takahashi K., Silva RLe Hylton D., Rudge J.S., Wiegand S.J., Campochiaro P.A. (2003). VEGF-TRAPR1R2 suppresses choroidal neovascularization and VEGF-induced breakdown of the blood–retinal barrier. J. Cell. Physiol..

[15] Lichtlen P., Lam T.T., Nork T.M., Streit T., Urech D.M. (2010). Relative contribution of VEGF and TNF-α in the cynomolgus laser-induced CNV model: Comparing the efficacy of bevacizumab, adalimumab, and ESBA105. Investig. Ophthalmol. Vis. Sci..

[16] Volland S., Esteve-Rudd J., Hoo J., Yee C., Williams D.S. (2015). A comparison of some organizational characteristics of the mouse central retina and the human macula. PLoS ONE.

[17] Marmorstein A.D., Marmorstein L.Y. (2007). The challenge of modeling macular degeneration in mice. Trends Genet..

[18] Lambert V., Lecomte J., Hansen S., Blacher S., Gonzalez M.-L.A., Struman I., Sounni N.E., Rozet E., de Tullio P., Foidart J.M. (2013). Laser-induced choroidal neovascularization model to study age-related macular degeneration in mice. Nat. Protoc..

[19] Johnson L.V., Forest D.L., Banna C.D., Radeke C.M., Maloney M.A., Hu J., Spencer C.N., Walker A.M., Tsie M.S., Bok D. (2011). Cell culture model that mimics drusen formation and triggers complement activation associated with age-related macular degeneration. Proc. Natl. Acad. Sci. USA.

[20] Rabin D.M., Rabin R.L., Blenkinsop T.A., Temple S., Stern J.H. (2013). Chronic oxidative stress upregulates Drusen-related protein expression in adult human RPE stem cell-derived RPE cells: A novel culture model for dry AMD. Aging.

[21] Galloway C.A., Dalvi S., Hung S.S., MacDonald L.A., Latchney L.R., Wong R.C., Guymer R.H., Mackey D.A., Williams D.S., Chung M.M. (2017). Drusen in patient-derived hiPSC-RPE models of macular dystrophies. Proc. Natl. Acad. Sci. USA.

[22] Mullins R.F., Khanna A., Schoo D.P., Tucker B.A., Sohn E.H., Drack A.V., Stone E.M. (2014). Is age-related macular degeneration a microvascular disease?. Adv. Exp. Med. Biol..

[23] Sho K., Takahashi K., Yamada H., Wada M., Nagai Y., Otsuji T., Nishikawa M., Mitsuma Y., Yamazaki Y., Matsumura M. (2003). Polypoidal choroidal vasculopathy: Incidence, demographic features, and clinical characteristics. Arch. Ophthalmol..

[24] Okubo A., Sameshima M., Uemura A., Kanda S., Ohba N. (2002). Clinicopathological correlation of polypoidal choroidal vasculopathy revealed by ultrastructural study. Br. J. Ophthalmol..

[25] Yuzawa M., Mori R., Kawamura A. (2005). The origins of polypoidal choroidal vasculopathy. Br. J. Ophthalmol..

[26] Fei P., Zaitoun I., Farnoodian M., Fisk D.L., Wang S., Sorenson C.M., Sheibani N. (2014). Expression of thrombospondin-1 modulates the angioinflammatory phenotype of choroidal endothelial cells. PLoS ONE.

[27] Geisen P., McColm J.R., Hartnett M.E. (2006). Choroidal endothelial cells transmigrate across the retinal pigment epithelium but do not proliferate in response to soluble vascular endothelial growth factor. Exp. Eye Res..

[28] Wang H., Hartnett M.E. (2016). Regulation of signaling events involved in the pathophysiology of neovascular AMD. Mol. Vis..

[29] Songstad A.E., Worthington K.S., Chirco K.R., Giacalone J.C., Whitmore S.S., Anfinson K.R., Ochoa D., Cranston C.M., Riker M.J., Neiman M. (2017). Connective Tissue Growth Factor Promotes Efficient Generation of Human Induced Pluripotent Stem Cell-Derived Choroidal Endothelium. STEM CELLS Transl. Med..

[30] Giacalone J.C., Miller M.J., Workalemahu G., Reutzel A.J., Ochoa D., Whitmore S.S., Stone E.M., Tucker B.A., Mullins R.F. (2019). Generation of an immortalized human choroid endothelial cell line (iChEC-1) using an endothelial cell specific promoter. Microvasc. Res..

[31] Wu K.X., Yeo N.J.Y., Ng C.Y., Chioh F.W.J., Fan Q., Tian X., Yang B., Narayanan G., Tay H.M., Hou H.W. (2022). Hyaluronidase-1-mediated glycocalyx impairment underlies endothelial abnormalities in polypoidal choroidal vasculopathy. BMC Biol..

[32] Ng C.-Y., Lee K.L., Muthiah M.D., Wu K.X., Chioh F.W.J., Tan K., Soon G.S.T., Shabbir A., Loo W.M., Low Z.S. (2022). Endothelial-immune crosstalk contributes to vasculopathy in nonalcoholic fatty liver disease. EMBO Rep..

[33] Chang Milbauer L., Wei P., Enenstein J., Jiang A., Hillery C.A., Scott J.P., Nelson S.C., Bodempudi V., Topper J.N., Yang R.B. (2008). Genetic endothelial systems biology of sickle stroke risk. Blood.

[34] Ecklu-Mensah G., Olsen R.W., Bengtsson A., Ofori M.F., Hviid L., Jensen A.T.R., Adams Y. (2018). Blood outgrowth endothelial cells (BOECs) as a novel tool for studying adhesion of Plasmodium falciparum-infected erythrocytes. PLoS ONE.

[35] Wei P., Milbauer L.C., Enenstein J., Nguyen J., Pan W., Hebbel R.P. (2011). Differential endothelial cell gene expression by African Americans versus Caucasian Americans: A possible contribution to health disparity in vascular disease and cancer. BMC Med..

[36] Benedito R., Roca C., Sörensen I., Adams S., Gossler A., Fruttiger M., Adams R.H. (2009). The notch ligands Dll4 and Jagged1 have opposing effects on angiogenesis. Cell.

[37] Gerhardt H., Golding M., Fruttiger M., Ruhrberg C., Lundkvist A., Abramsson A., Jeltsch M., Mitchell C., Alitalo K., Shima D. (2003). VEGF guides angiogenic sprouting utilizing endothelial tip cell filopodia. J. Cell Biol..

[38] Eilken H.M., Adams R.H. (2010). Dynamics of endothelial cell behavior in sprouting angiogenesis. Curr. Opin. Cell Biol..

[39] Jakobsson L., Franco C.A., Bentley K., Collins R.T., Ponsioen B., Aspalter I.M., Rosewell I., Busse M., Thurston G., Medvinsky A. (2010). Endothelial cells dynamically compete for the tip cell position during angiogenic sprouting. Nat. Cell Biol..

[40] Bentley K., Franco C.A., Philippides A., Blanco R., Dierkes M., Gebala V., Stanchi F., Jones M., Aspalter I.M., Cagna G. (2014). The role of differential VE-cadherin dynamics in cell rearrangement during angiogenesis. Nat. Cell Biol..

[41] Mittal B., Mishra A., Srivastava A., Kumar S., Garg N., Makowski G.S. (2014). Chapter One-Matrix Metalloproteinases in Coronary Artery Disease. Advances in Clinical Chemistry.

[42] Wu G., Luo J., Rana J.S., Laham R., Sellke F.W., Li J. (2006). Involvement of COX-2 in VEGF-induced angiogenesis via P38 and JNK pathways in vascular endothelial cells. Cardiovasc. Res..

[43] Gately S., Li W.W. (2004). Multiple roles of COX-2 in tumor angiogenesis: A target for antiangiogenic therapy. Semin. Oncol..

[44] Phelan M.W., Forman L.W., Perrine S.P., Faller D.V. (1998). Hypoxia increases thrombospondin-1 transcript and protein in cultured endothelial cells. J. Lab. Clin. Med..

[45] Labrousse-Arias D., Castillo-González R., Rogers N.M., Torres-Capelli M., Barreira B., Aragonés J., Cogolludo Á., Isenberg J.S., Calzada M.J. (2016). HIF-2α-mediated induction of pulmonary thrombospondin-1 contributes to hypoxia-driven vascular remodelling and vasoconstriction. Cardiovasc. Res..

[46] Salani D., Taraboletti G., Rosanò L., Di Castro V., Borsotti P., Giavazzi R., Bagnato A. (2000). Endothelin-1 induces an angiogenic phenotype in cultured endothelial cells and stimulates neovascularization in vivo. Am. J. Pathol..

[47] Li Z., Solomonidis E.G., Meloni M., Taylor R.S., Duffin R., Dobie R., Magalhaes M.S., Henderson B.E.P., Louwe P.A., D’Amico G. (2019). Single-cell transcriptome analyses reveal novel targets modulating cardiac neovascularization by resident endothelial cells following myocardial infarction. Eur. Heart J..

[48] Kouprina N., Pavlicek A., Collins N.K., Nakano M., Noskov V.N., Ohzeki J.-I., Mochida G.H., Risinger J.I., Goldsmith P., Gunsior M. (2005). The microcephaly ASPM gene is expressed in proliferating tissues and encodes for a mitotic spindle protein. Hum. Mol. Genet..

[49] Kang H.J., Jung S.K., Kim S.J., Chung S.J. (2008). Structure of human alpha-enolase (hENO1), a multifunctional glycolytic enzyme. Acta Crystallogr. D Biol. Crystallogr..

[50] Orosz F., Oláh J., Ovádi J. (2009). Triosephosphate isomerase deficiency: New insights into an enigmatic disease. Biochim. Biophys. Acta (BBA)-Mol. Basis Dis..

[51] Dumas S.J., García-Caballero M., Carmeliet P. (2020). Metabolic Signatures of Distinct Endothelial Phenotypes. Trends Endocrinol. Metab..

[52] Yetkin-Arik B., Vogels I.M.C., Neyazi N., van Duinen V., Houtkooper R.H., van Noorden C.J.F., Klaassen I., Schlingemann R.O. (2019). Endothelial tip cells in vitro are less glycolytic and have a more flexible response to metabolic stress than non-tip cells. Sci. Rep..

[53] Atkinson S.P., Collin J., Irina N., Anyfantis G., Kyung B.K., Lako M., Armstrong L. (2012). A Putative Role for the Immunoproteasome in the Maintenance of Pluripotency in Human Embryonic Stem Cells. STEM CELLS.

[54] Wäsch R., Engelbert D. (2005). Anaphase-promoting complex-dependent proteolysis of cell cycle regulators and genomic instability of cancer cells. Oncogene.

[55] Colombo S.L., Palacios-Callender M., Frakich N., De Leon J., Schmitt C.A., Boorn L., Davis N., Moncada S. (2010). Anaphase-promoting complex/cyclosome-Cdh1 coordinates glycolysis and glutaminolysis with transition to S phase in human T lymphocytes. Proc. Natl. Acad. Sci. USA.

[56] Almeida A., Bolaños J.P., Moncada S. (2010). E3 ubiquitin ligase APC/C-Cdh1 accounts for the Warburg effect by linking glycolysis to cell proliferation. Proc. Natl. Acad. Sci. USA.

[57] Kempe S., Kestler H., Lasar A., Wirth T. (2005). NF-kappaB controls the global pro-inflammatory response in endothelial cells: Evidence for the regulation of a pro-atherogenic program. Nucleic Acids Res..

[58] Sainson R.C.A., Johnston D.A., Chu H.C., Holderfield M.T., Nakatsu M.N., Crampton S.P., Davis J., Conn E., Hughes C.C.W. (2008). TNF primes endothelial cells for angiogenic sprouting by inducing a tip cell phenotype. Blood.

[59] Wang D., Anderson J.C., Gladson C.L. (2005). The Role of the Extracellular Matrix in Angiogenesis in Malignant Glioma Tumors. Brain Pathol..

[60] Senger D.R., Perruzzi C.A., Streit M., Koteliansky V.E., de Fougerolles A.R., Detmar M. (2002). The alpha(1)beta(1) and alpha(2)beta(1) integrins provide critical support for vascular endothelial growth factor signaling, endothelial cell migration, and tumor angiogenesis. Am. J. Pathol..

[61] Friedlander M., Brooks P.C., Shaffer R.W., Kincaid C.M., Varner J.A., Cheresh D.A. (1995). Definition of Two Angiogenic Pathways by Distinct αv Integrins. Science.

[62] Reynolds L.E., Wyder L., Lively J.C., Taverna D., Robinson S.D., Huang X., Sheppard D., Hynes R.O., Hodivala-Dilke K.M. (2002). Enhanced pathological angiogenesis in mice lacking β3 integrin or β3 and β5 integrins. Nat. Med..

[63] Nassar K., Grisanti S., Elfar E., Lüke J., Lüke M., Grisanti S. (2015). Serum cytokines as biomarkers for age-related macular degeneration. Graefe’s Arch. Clin. Exp. Ophthalmol..

[64] Fu B., Liu Z.L., Zhang H., Gu F. (2017). Interleukin-13 and age-related macular degeneration. Int. J. Ophthalmol..

[65] Baba T., Miyazaki D., Inata K., Uotani R., Miyake H., Sasaki S.-I., Shimizu Y., Inoue Y., Nakamura K. (2020). Role of IL-4 in bone marrow driven dysregulated angiogenesis and age-related macular degeneration. eLife.

[66] Fukushi J.-I., Ono M., Morikawa W., Iwamoto Y., Kuwano M. (2000). The Activity of Soluble VCAM-1 in Angiogenesis Stimulated by IL-4 and IL-13. J. Immunol..

[67] Simons M., Gordon E., Claesson-Welsh L. (2016). Mechanisms and regulation of endothelial VEGF receptor signalling. Nat. Rev. Mol. Cell Biol..

[68] Héroult M., Schaffner F., Augustin H.G. (2006). Eph receptor and ephrin ligand-mediated interactions during angiogenesis and tumor progression. Exp. Cell Res..

[69] Vreeken D., Zhang H., van Zonneveld A.J., van Gils J.M. (2020). Ephs and Ephrins in Adult Endothelial Biology. Int. J. Mol. Sci..

[70] Wiedemann E., Jellinghaus S., Ende G., Augstein A., Sczech R., Wielockx B., Weinert S., Strasser R.H., Poitz D.M. (2017). Regulation of endothelial migration and proliferation by ephrin-A1. Cell Signal.

[71] Longchamp A., Mirabella T., Arduini A., MacArthur M.R., Das A., Treviño-Villarreal J.H., Hine C., Ben-Sahra I., Knudsen N.H., Brace L.E. (2018). Amino Acid Restriction Triggers Angiogenesis via GCN2/ATF4 Regulation of VEGF and H(2)S Production. Cell.

[72] Oberkersch R.E., Santoro M.M. (2019). Role of amino acid metabolism in angiogenesis. Vasc. Pharmacol..

[73] Rohlenova K., Goveia J., García-Caballero M., Subramanian A., Kalucka J., Treps L., Falkenberg K.D., de Rooij L.P.M.H., Zheng Y., Lin L. (2020). Single-Cell RNA Sequencing Maps Endothelial Metabolic Plasticity in Pathological Angiogenesis. Cell Metab..

[74] Hneino M., Blirando K., Buard V., Tarlet G., Benderitter M., Hoodless P., François A., Milliat F. (2012). The TG-interacting factor TGIF1 regulates stress-induced proinflammatory phenotype of endothelial cells. J. Biol. Chem..

[75] O’Reilly M.S., Boehm T., Shing Y., Fukai N., Vasios G., Lane W.S., Flynn E., Birkhead J.R., Olsen B.R., Folkman J. (1997). Endostatin: An Endogenous Inhibitor of Angiogenesis and Tumor Growth. Cell.

[76] Seppinen L., Pihlajaniemi T. (2011). The multiple functions of collagen XVIII in development and disease. Matrix Biol..

[77] Elamaa H., Sormunen R., Rehn M., Soininen R., Pihlajaniemi T. (2005). Endostatin Overexpression Specifically in the Lens and Skin Leads to Cataract and Ultrastructural Alterations in Basement Membranes. Am. J. Pathol..

[78] Edgar L.T., Underwood C.J., Guilkey J.E., Hoying J.B., Weiss J.A. (2014). Extracellular Matrix Density Regulates the Rate of Neovessel Growth and Branching in Sprouting Angiogenesis. PLoS ONE.

[79] Davis G.E., Senger D.R. (2005). Endothelial Extracellular Matrix. Circ. Res..

[80] Rondaij M.G., Bierings R., Kragt A., van Mourik J.A., Voorberg J. (2006). Dynamics and plasticity of Weibel-Palade bodies in endothelial cells. Arter. Thromb. Vasc. Biol..

[81] Yamashita M., Matsumoto M., Hayakawa M., Sakai K., Fujimura Y., Ogata N. (2018). Intravitreal injection of aflibercept, an anti-VEGF antagonist, down-regulates plasma von Willebrand factor in patients with age-related macular degeneration. Sci. Rep..

[82] Lip P.-L., Blann A.D., Hope-Ross M., Gibson J.M., Lip G.Y.H. (2001). Age-related macular degeneration is associated with increased vascular endothelial growth factor, hemorheology and endothelial dysfunction. Ophthalmology.

[83] Gampel A., Moss L., Jones M.C., Brunton V., Norman J.C., Mellor H. (2006). VEGF regulates the mobilization of VEGFR2/KDR from an intracellular endothelial storage compartment. Blood.

[84] Tan W., Zou J., Yoshida S., Jiang B., Zhou Y. (2020). The Role of Inflammation in Age-Related Macular Degeneration. Int. J. Biol. Sci..

[85] Edwards A.O., Ritter R., Abel K.J., Manning A., Panhuysen C., Farrer L.A. (2005). Complement Factor H Polymorphism and Age-Related Macular Degeneration. Science.

[86] Scimone C., Donato L., Alibrandi S., Esposito T., Alafaci C., D’Angelo R., Sidoti A. (2020). Transcriptome analysis provides new molecular signatures in sporadic Cerebral Cavernous Malformation endothelial cells. Biochim. Biophys. Acta Mol. Basis Dis..

[87] Scimone C., Donato L., Alafaci C., Granata F., Rinaldi C., Longo M., D’Angelo R., Sidoti A. (2020). High-Throughput Sequencing to Detect Novel Likely Gene-Disrupting Variants in Pathogenesis of Sporadic Brain Arteriovenous Malformations. Front. Genet..

[88] Scimone C., Donato L., Marino S., Alafaci C., D’Angelo R., Sidoti A. (2019). Vis-à-vis: A focus on genetic features of cerebral cavernous malformations and brain arteriovenous malformations pathogenesis. Neurol. Sci..

[89] Voronov E., Carmi Y., Apte R.N. (2014). The role IL-1 in tumor-mediated angiogenesis. Front. Physiol..

[90] Okamoto H., Yoshimatsu Y., Tomizawa T., Kunita A., Takayama R., Morikawa T., Komura D., Takahashi K., Oshima T., Sato M. (2019). Interleukin-13 receptor α2 is a novel marker and potential therapeutic target for human melanoma. Sci. Rep..

[91] Geindreau M., Bruchard M., Vegran F. (2022). Role of Cytokines and Chemokines in Angiogenesis in a Tumor Context. Cancers.

[92] Paschalaki K.E., Randi A.M. (2018). Recent Advances in Endothelial Colony Forming Cells Toward Their Use in Clinical Translation. Front. Med..

[93] Paschalaki K.E., Starke R.D., Hu Y., Mercado N., Margariti A., Gorgoulis V.G., Randi A.M., Barnes P.J. (2013). Dysfunction of Endothelial Progenitor Cells from Smokers and Chronic Obstructive Pulmonary Disease Patients Due to Increased DNA Damage and Senescence. Stem Cells.

[94] Ormiston M.L., Toshner M.R., Kiskin F.N., Huang C.J., Groves E., Morrell N.W., Rana A.A. (2015). Generation and Culture of Blood Outgrowth Endothelial Cells from Human Peripheral Blood. J. Vis. Exp..

[95] Nakatsu M.N., Davis J., Hughes C.C. (2007). Optimized fibrin gel bead assay for the study of angiogenesis. J. Vis. Exp..

[96] Hao Y., Hao S., Andersen-Nissen E., Mauck W.M., Zheng S., Butler A., Lee M.J., Wilk A.J., Darby C., Zager M. (2021). Integrated analysis of multimodal single-cell data. Cell.

[97] Stuart T., Butler A., Hoffman P., Hafemeister C., Papalexi E., Mauck W.M., Hao Y., Stoeckius M., Smibert P., Satija R. (2019). Comprehensive Integration of Single-Cell Data. Cell.

[98] McGinnis C.S., Murrow L.M., Gartner Z.J. (2019). DoubletFinder: Doublet Detection in Single-Cell RNA Sequencing Data Using Artificial Nearest Neighbors. Cell Syst..

[99] Lukowski S.W., Patel J., Andersen S.B., Sim S.L., Wong H.Y., Tay J., Winkler I., Powell J.E., Khosrotehrani K. (2019). Single-Cell Transcriptional Profiling of Aortic Endothelium Identifies a Hierarchy from Endovascular Progenitors to Differentiated Cells. Cell Rep..

[100] Zappia L., Oshlack A. (2018). Clustering trees: A visualization for evaluating clusterings at multiple resolutions. GigaScience.

[101] Yu G., Wang L.G., Han Y., He Q.Y. (2012). clusterProfiler: An R package for comparing biological themes among gene clusters. OMICS.

[102] Yu G., He Q.-Y. (2016). ReactomePA: An R/Bioconductor package for reactome pathway analysis and visualization. Mol. BioSyst..

