# Supplementary material for: Single-Cell Transcriptome of Wet AMD Patient-Derived Endothelial Cells in Angiogenic Sprouting
Source: Int J Mol Sci. 2022 Oct 19;23(20):12549. doi: 10.3390/ijms232012549 (PMC9604336; doi:10.3390/ijms232012549)
Supplement: Supplementary file 1 [file ijms-23-12549-s001.zip › ijms-1958261-supplementary.pdf]

Supplemental Materials

Supplemental Figure S1

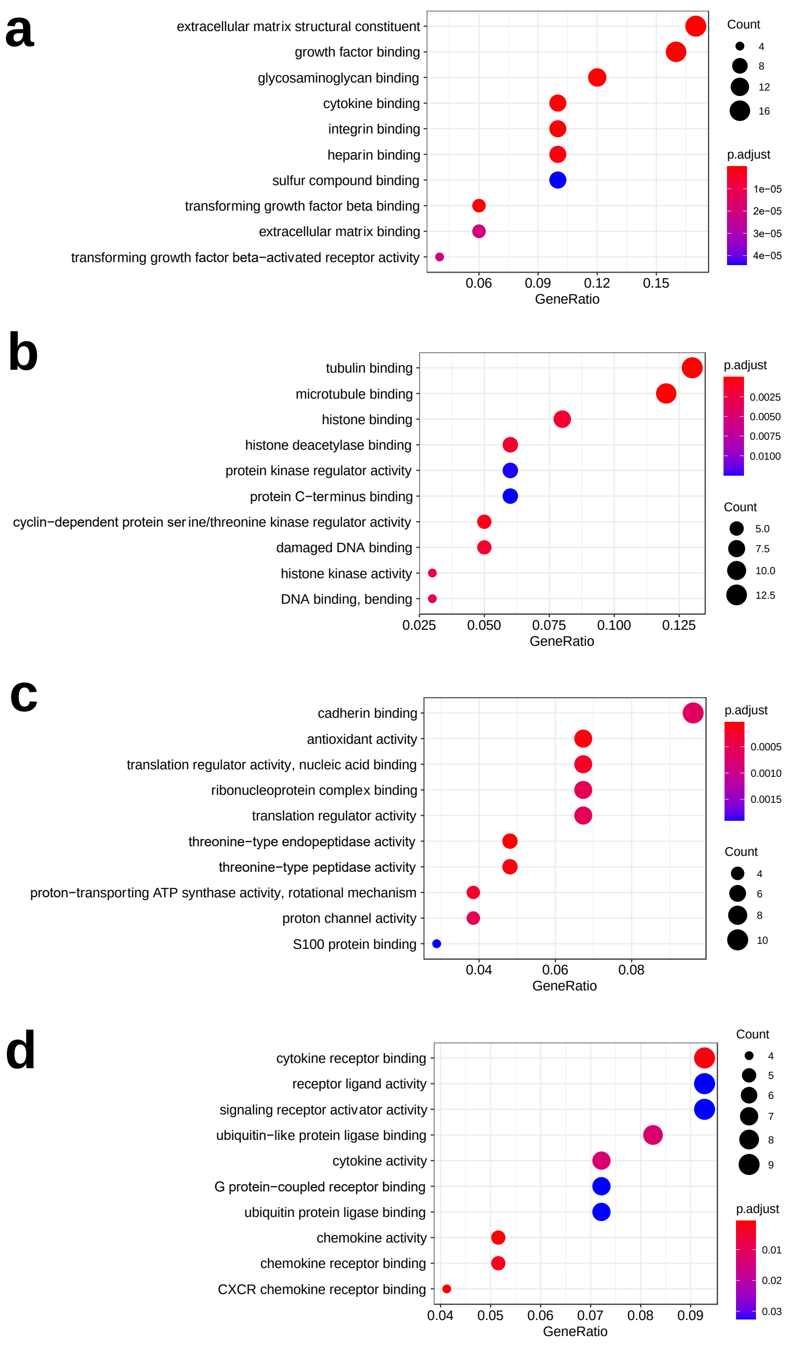

Supplemental Figure S1: Gene ontology molecular function analysis of cluster markers for each BOEC subpopulation. (a) 'Vascular remodeling' population; **(b)** 'Proliferative' population; **(c)** 'Energetically primed' population; **(d)** 'Activated' population. The top 10 enriched processes based on adjusted p values (p.adjust) are shown, with 'count' indicating the number of genes underlying each term.

Supplemental Table S1: Demographics of wet AMD and normal donors from whom blood outgrowth endothelial cells (BOECs) were derived.

Disease status	Age	Genotypes		
		ARMS2, rs10490924 (risk allele: T)	HTRA1, rs11200638 (risk allele: A)	CFH, rs800292 (risk allele: G)
Wet AMD	71	TT	AA	GA
	86	TT	AA	AA
Normal control	46	GG	GG	GA

Additional Table S2: Key resources used in this study.

REAGENT or RESOURCE	SOURCE	IDENTIFIER
**Biological samples**		
PBMC	This paper	N/A
**Chemicals, peptides, and recombinant proteins**		
TRITC-Phalloidin	Merck	90228
**Critical commercial assays**		
Chromium Single Cell 3' v3 Reagent Kit	10X Genomics	PN-1000075
**Experimental models: Cell lines**		
Patient-derived BOECs	This paper	N/A
**Software and algorithms**		
Seurat v 4.1.1	References 91 and 92 in Main Manuscript	https://satijalab.org/seurat/
clusterProfiler v 4.2.2	Reference 96 in Main Manuscript	https://github.com/YuLab-SMU/clusterProfiler
ReactomePA v 1.38.0	Reference 97 in Main Manuscript	https://github.com/YuLab-SMU/ReactomePA
ZEN BLUE	ZEISS	https://www.zeiss.com/microscopy/int/products/microscope-software/zen.html
**Others**		
Confocal Airyscan Microscope LSM800	ZEISS	N/A
